# Effects of Lipopolysaccharides from *Hafnia alvei* PCM1200, *Proteus penneri* 12, and *Proteus vulgaris* 9/57 on Liposomal Membranes Composed of Natural Egg Yolk Lecithin (EYL) and Synthetic DPPC: An EPR Study and Computer Simulations

**DOI:** 10.3390/membranes16010038

**Published:** 2026-01-08

**Authors:** Dariusz Man, Barbara Pytel, Izabella Pisarek

**Affiliations:** 1 Institute of Physics, Opole University, Oleska 48, 45-052 Opole, Poland; 2 Department of Land Protection and Spatial Management, Institute of Environmental Engineering and Biotechnology, Opole University, kard. B. Kominka 6, 45-032 Opole, Poland; izabella.pisarek@uni.opole.pl

**Keywords:** LPS, liposomal membranes, monte carlo simulation, EPR spectroscopy, spin probes

## Abstract

The aim of this study was to investigate the effects of three lipopolysaccharides (LPS), obtained from *Hafnia alvei* PCM 1200, *Proteus penneri* 12, and *Proteus vulgaris* 9/57, on the fluidity of liposomal lipid membranes. The experiments were performed on liposomes composed of egg yolk lecithin (EYL) in the liquid-crystalline phase and synthetic lecithin (DPPC) in the gel phase. The experimental results were compared with data obtained from a computational model of the membrane surface layer. Membrane fluidity was assessed using EPR spectroscopy with the spin probes TEMPO (surface layer; changes in the F parameter) and 16-DOXYL (hydrophobic core; changes in the *τ* parameter). In EYL liposomes, all LPS samples induced a reduction in surface-layer fluidity (decrease in the F/F_0_ ratio). In contrast, effects on the hydrophobic core (*τ*/*τ*_0_) were observed only at low dopant concentrations (<0.2%), above which membrane fluidity plateaued. In DPPC membranes, the response was more complex: local minima in F/F_0_ and maxima in *τ*/*τ*_0_ were detected, indicating transient alterations in membrane stiffening and plasticization that depended on the specific LPS applied. Computational simulations of the membrane surface further confirmed the greater susceptibility of low-mobility systems (corresponding to the gel phase) to dopant-induced perturbations. In the model, the best agreement with the EPR data was obtained when an effective dopant charge of *q* = 3 was assumed.

## 1. Introduction

Biological membranes represent fundamental cellular structures composed primarily of a lipid bilayer, into which proteins, cholesterol, and carbohydrates are incorporated. They perform essential functions such as protection—by separating the cell from its external environment—regulation of substance transport, compartmentalization of intracellular space, and signal transduction. Owing to their selective permeability, membranes enable the cell to maintain optimal conditions for physiological processes. Since natural biological membranes are highly complex structures, their precise mathematical modeling is extremely challenging and often difficult to interpret. A suitable experimental model for studying such systems is provided by liposomes, whose formation process can be precisely controlled. Changes in their fluidity can be investigated using electron paramagnetic resonance (EPR) spectroscopy, which employs appropriately selected spin probes [1,2,3,4,5,6,7]. One of the methods for producing liposomes from lecithin is sonication, which allows the preparation of unilamellar liposomes with a diameter of approximately 200 nm [8,9,10,11]. An advantage of using liposomes in research is the ability to control their molecular composition—a feature that is difficult to achieve in natural biological membranes. In the present study, the effect of three selected groups of lipopolysaccharides (LPS), of medical relevance, on the fluidity of liposomal membranes was investigated. These compounds—LPS from *Hafnia alvei* PCM 1200, *Proteus penneri* 12, and *Proteus vulgaris* 9/57—were extracted from Gram-negative bacteria by researchers from the Department of Immunochemistry at the Ludwik Hirszfeld Institute of Immunology and Experimental Therapy, Polish Academy of Sciences.

*Hafnia alvei* PCM 1200 is a commensal Gram-negative bacterium belonging to the *Enterobacteriaceae* family. It has been isolated from a variety of sources, including the intestinal tracts of humans and animals, as well as from soil, water, and food products [12,13,14]. This strain has been previously studied for the structural and immunochemical properties of its lipopolysaccharides [12].

*Proteus penneri* 12 represents a clinical isolate of the *Proteus* genus, typically recovered from human urine, wound exudates, and other pathological materials [15,16,17]. Environmental isolates of *Proteus* species are also common in soil and wastewater. LPS from *P. penneri* 12 has been analyzed in studies classifying its core region and O-antigen structures [16].

*Proteus vulgaris* 9/57 is another representative of the *Proteus* genus, frequently found in the intestinal flora of humans and animals, as well as in environmental niches such as water, sewage, and soil [18,19,20]. Clinically, *P. vulgaris* is associated with urinary tract and wound infections, and its LPS structure serves as an important antigenic determinant in pathogenicity research [19].

At room temperature, DPPC lecithin is in the gel phase, the main phase-transition temperature being approximately 41 °C [21,22]. In contrast, EYL lecithin at room temperature is in the liquid-crystalline phase and its main phase-transition temperature is significantly lower than that of DPPC [23,24]. Liposomes composed of lecithin are diamagnetic; therefore, EPR must be employed with spin probes. The spin probe 16-DOXYL is a fatty-acid derivative and localizes in the membrane similarly to phospholipid molecules. Hence, 16-DOXYL is commonly used in EPR experiments with lipid membranes. The spin probe TEMPO dissolves both in the hydrophobic region of the membrane and in the aqueous environment. Therefore, it may be used to determine changes occurring at the water–lipid phase boundary. Depending on the degree of membrane fluidity, it will preferentially reside in the aqueous environment (rigid membrane) or in the lipid environment (membrane of increased fluidity). Additionally, this study employed computer simulations of the lipid membrane surface. The simulation model represented the membrane surface layer as an array of electric dipoles, which were free to rotate along their symmetry axes and translate within the XY plane. The software allowed precise control over membrane fluidity, as well as the size and charge of introduced impurities. This simulation framework had been previously utilized in several studies by the authors [25,26,27]. In the present work, minor modifications were introduced to the program, enabling simulations of larger dipole arrays (44 × 44 = 1936 dipoles) and impurities up to 4 nm in size, under both liquid-crystalline and gel-phase membrane conditions. The main computational algorithms of the program were described in detail in ref. [26].

## 2. Materials and Methods

### 2.1. LPS Preparation

**Figure 1:**
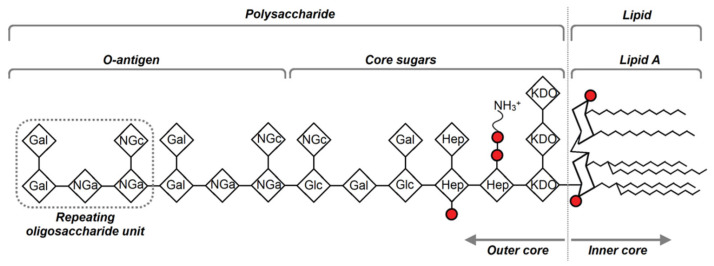
Schematic representation of the LPS molecule with its major functional regions indicated, red circles indicate phosphate groups.

LPS of LPS1 = *H. alvei* PCM 1200, LPS2 = *P. penneri* 12 and LPS3 = *P. vulgaris* 9/57 were kindly provided by Prof. Czesław Lugowski from the Department of Immunochemistry, Ludwik Hirszfeld Institute of Immunology and Experimental Therapy, Polish Academy of Sciences, Wrocław, Poland (Figure 1).

### 2.2. Lecithin and Spin Probes

**Figure 2:**
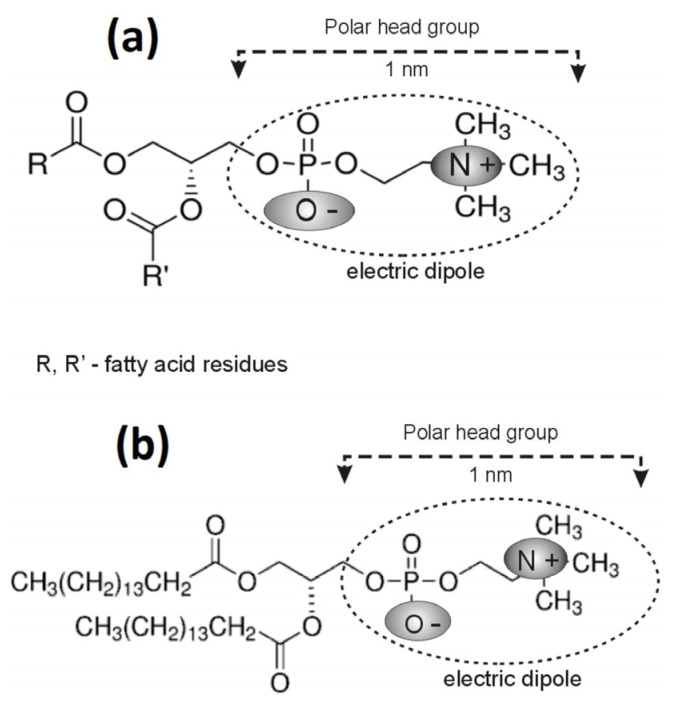
Chemical structures of lecithin molecules: (**a**) egg yolk lecithin (EYL) and (**b**) synthetic lecithin (DPPC).

Egg yolk lecithin (EYL) L-α-Lecithin and Synthetic lecithin (DPPC) 1,2-Dipalmitoyl-sn-glycero-3-phosphocholine were purchased from Sigma-Aldrich, Poznań, Poland (Figure 2).

**Figure 3:**
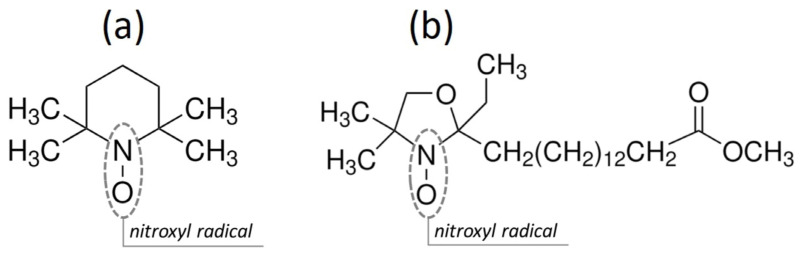
The structures of spin labels: (**a**) TEMPO, (**b**) 16-DOXYL stearic acid methyl ester.

Two different spin labels (Figure 3) purchased from Sigma-Aldrich, Poznań, Poland were used in EPR tests. The first was a 2,2,6,6-tetramethylpiperidine-1-oxyl (TEMPO), empirical formula (Hill Notation) C9H18NO, molecular weight 156.25 (Figure 3a). The structures of spin labels: (a) TEMPO, (b) 16-DOXYL stearic acid methyl ester. The second was a 2-ethyl-2-(15-methoxy-15-oxopentadecyl)-4,4-dimethyl-3-oxazolidinyloxy (16-DOXYL), empirical formula (Hill Notation) C23H44NO4, molecular weight 398.60 (Figure 3b).

### 2.3. EPR Measurements

**Figure 4:**
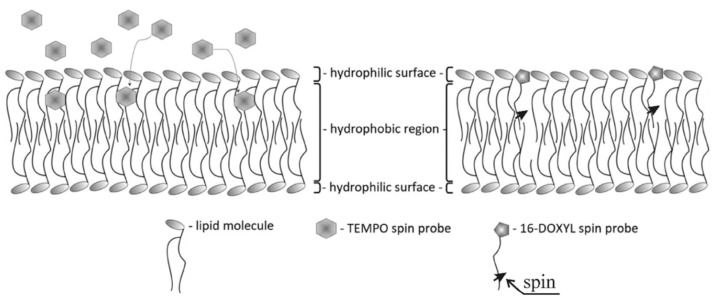
Schematic illustration depicting the spatial localization of spin probes within the lipid bilayer.

To evaluate the effect of LPS dopants on liposome membrane fluidity, electron paramagnetic resonance (EPR) spectroscopy was employed, using spin probes as molecular reporters of membrane dynamics. Two spin probes were selected: 16-DOXYL-stearic acid methyl ester (16-DOXYL), which localizes in the hydrophobic core of the membrane, and TEMPO, which resides closer to the membrane surface (Figure 4). These probes are well known for their sensitivity to changes in membrane fluidity at distinct depths within the lipid bilayer.

Liposome preparation. Liposomes were prepared by sonication of egg yolk lecithin (EYL) and synthetic lecithin (DPPC) in distilled water. A predetermined amount of lecithin solution in chloroform was transferred to a quartz vessel, and the solvent was completely evaporated under vacuum to form a thin lipid film. Subsequently, distilled water was added, and the suspension was subjected to sonication using an ultrasonic disintegrator (UD-20; Techpan, Warsaw, Poland) with alternating cycles of 20 s sonication and 30 s cooling to prevent overheating, for a total duration of 5 min. This procedure yielded large unilamellar vesicles (LUVs). Each sample contained 60 mM EYL lecithin in 1.5 mL of water. Following sonication, the resulting liposome suspension was divided into three portions, and spin probes (TEMPO or 16-DOXYL) were added at a concentration of 350 ppm relative to the lipid content. The samples were thoroughly mixed and allowed to equilibrate for 10 min to ensure uniform distribution of the spin probes within the lipid bilayer.

EPR spectra were recorded at a constant temperature of 22 °C using an MX-201R spectrometer (TU Wrocław, Wrocław, Poland) under the following operating conditions: microwave power P = 60 mW, sweep range ΔB = 7 mT, modulation amplitude δB = 0.1 mT, time constant *τ* = 0.1 s, and sweep time t = 68 s. Samples were loaded into thin-walled glass capillaries (1 mm diameter). Measurements were first performed for pure liposomes, followed by samples with increasing concentrations of admixtures (corresponding LPSs), added incrementally by 0.05% up to 0.5% and by 0.1% up to 1%. This procedure was repeated for all tested LPSs in membranes composed of EYL and DPPC, using both TEMPO and 16-DOXYL probes. Each measurement was repeated three times, and the final spectra were averaged automatically by the spectrometer software, Version 3.24. The spectroscopic parameter F for the TEMPO probe was determined with an accuracy of ±2%, while the rotational correlation time *τ* for the 16-DOXYL probe was determined with an accuracy of ±3%.

### 2.4. Computer-Based Model

**Figure 5:**
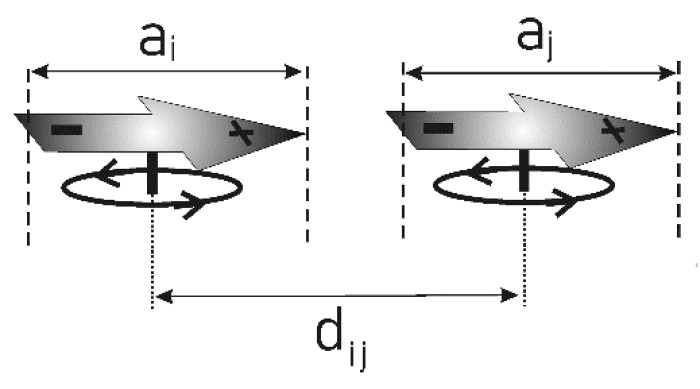
Schematic representation of the configuration of two dipoles embedded in a matrix in a computer simulation, showing the corresponding charge distribution and geometric parameters.

To gain a deeper understanding of the processes occurring at the membrane surface, a computer-based model was developed. The model comprises a system of *N* electric dipoles, each capable of rotating about its own axis—perpendicular to the membrane surface—and translating within the membrane’s XY plane. The total energy of the surface-layer system (Hamiltonian) can be expressed as follows:

(1) $$H=\sum_{\left(i\right)}\frac{p_{i}^{2}}{2m}+\sum_{\left(i\right)}\frac{L_{i}^{2}}{2I}+\sum_{\left(i<j\right)}U_{ij}$$

where *m* denotes the mass of a dipole and *I* its moment of inertia. The first two terms correspond to the kinetic energy associated with the translational and rotational motion of the dipoles (Figure 5), whereas the third term accounts for the Coulomb and Lennard–Jones interactions among all dipoles.

(2) $$U_{ij}=\frac{e^{2}}{4\pi \varepsilon_{0}\varepsilon_{r}}\left(\frac{1}{\left|{\vec{d}}_{ij}+\vec{a_{j}}-\vec{a_{i}}\right|}-\frac{1}{\left|\vec{d_{ij}}-\vec{a_{j}}-{\vec{a}}_{i}\right|}+\frac{1}{\left|\vec{d_{ij}}-\vec{a_{j}}+\vec{a_{i}}\right|}-\frac{1}{\left|\vec{d_{ij}}+\vec{a_{j}}+\vec{a_{i}}\right|}\right)+\;4\varepsilon \left[{\left(\frac{\sigma }{\left|{\vec{d}}_{ij}\right|}\right)}^{12}-{\left(\frac{\sigma }{\left|{\vec{d}}_{ij}\right|}\right)}^{6}\right]$$

The vectors *a* and *d*, shown in Figure 5, represent geometrical parameters of the dipoles.

Thus, the system possesses three degrees of freedom—one corresponding to rotational motion and two associated with translational motion. In a real membrane, these dipoles represent the polar head groups of lipid molecules, whose motion is significantly constrained by the attached hydrocarbon chains. The presence of these chains limits the ability of the head groups (dipoles) to approach one another. It is evident that the dynamics of the chains strongly influence the dynamics of the dipolar heads. In the present model, this influence is assumed to be purely stochastic and uncorrelated—an evident simplification. Conceptually, the system can be regarded as a set of dipoles rigidly attached to small circular “corks” floating on a lipid surface (Figure 5). In the model employed, the parameter defining the coupling between these degrees of freedom is denoted by *k* (defined later in the text). This parameter determines the ratio between the mean displacement of a dipole on the membrane surface and the angular change in its orientation during a single simulation step. The starting point for this definition is the principle of equipartition of energy, which states that the mean kinetic energy associated with rotational motion equals the mean kinetic energy associated with translational motion along each axis (*x* and *y*).

(3) $$\frac{\langle p_{x_{i}}^{2}\rangle }{2m}=\frac{\langle p_{y_{i}}^{2}\rangle }{2m}=\frac{\langle L_{i}^{2}\rangle }{2I}$$

Let *t* denote a single simulation time step. The mean translational displacements, *dx* and *dy*, as well as the mean angular displacement, *dα*, during this step can be expressed as follows:

(4) $$\langle dx\rangle =\frac{\langle p_{x}\rangle }{m}\;t$$

(5) $$\;\langle dy\rangle =\frac{\langle p_{y}\rangle }{m}\;t$$

(6) $$\;\langle d\alpha \rangle =\frac{\langle L\rangle }{I}\;t$$

Using classical relationships between momentum and angular momentum, the following relation between translational and rotational motion can be written:

(7) $$\langle {\left(\delta A\right)}^{2}\rangle =\langle A^{2}\rangle -{\langle A\rangle }^{2}$$

The impact of the surrounding aqueous environment [28] and head–chain interactions cannot be explicitly incorporated, as neither the chain dynamics nor their effects on lipid-head mobility are sufficiently characterized. In this study, these effects are assumed to be stochastic, potentially affecting the translational and rotational degrees of freedom to varying extents. Adopting the approach of [29,30,31], we define a dimensionless mobility parameter *k* that characterizes the relative magnitude of translational to rotational motion:

(8) $$k=\frac{\pi }{l}\;\frac{\langle dx\rangle }{\langle d\alpha \rangle }$$

where *l* denotes the dipole length. Low values of *k* effectively immobilize translational motion, resulting in domain-like end-states governed by dipole orientation. Conversely, high *k* values yield end-states marked by pronounced linear (chain-like) aggregation of dipoles. Experimentally, this parameter may be correlated with membrane viscosity. Each simulation is performed for a fixed parameter set (*k*, *T*). The initial configuration is generated either by random dipole placement or by loading a predefined state. The system evolves through a sequence of configurations (a Markov chain) according to the Metropolis algorithm [32,33]. Conceptually, this algorithm executes a random walk in configuration space biased towards states of lower total energy. At each step, a randomly selected dipole is rotated by a random angle and displaced by a random vector, while preserving the relationship defined by equation 8. This perturbation results in a variation in the total potential energy Δ*E_p_*. According to the Metropolis criterion, the new state is automatically accepted if Δ*E_p_* ≤ 0; otherwise, it is accepted with probability *P*:

(9) $$P=\exp\left(-\frac{\Delta E_{p}}{k_{B}T}\right)$$

where *k_B_* is the Boltzmann constant and *T* is the temperature. If the newly proposed state is rejected, the system reverts to the previous configuration. After a certain period, the system reaches equilibrium, characterized by energy fluctuations about a stable mean value.

## 3. Results and Discussion

### 3.1. EPR Experiment

To investigate the effect of the tested LPS admixtures on the membrane fluidity of liposomes, the electron paramagnetic resonance (EPR) spectroscopy technique was employed using appropriately selected spin probes. The analyzed LPS molecules shared a similar hydrophobic part (lipid A), but differed in their hydrophilic regions composed of the core oligosaccharide and O-antigen fragments (Figure 1).

#### 3.1.1. Rotational Correlation Time Analysis

**Figure 6:**
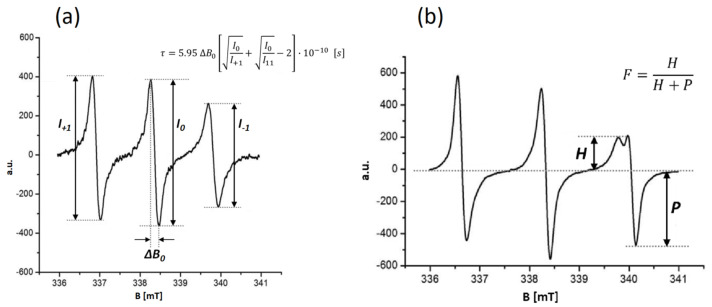
EPR spectra of spin probes incorporated into the liposome membrane. (**a**) 16-DOXYL stearic acid methyl ester probe and the corresponding rotational correlation time (*τ*) formula. (**b**) TEMPO probe and the spectroscopic partition parameter (F), where H denotes the signal from the probe in the lipid environment and P denotes the signal from the probe in the aqueous environment.

Figure 6a presents the dependence of the spectroscopic parameter—the rotational correlation time (*τ*)—obtained for the 16-DOXYL stearic acid methyl ester spin probe embedded in the hydrophobic region of the liposomal bilayer. The molecular structure of the probe ensures that its paramagnetic nitroxide group is located in the central region of the lipid bilayer. An increase in *τ* corresponds to a slower rotational motion of the probe about its symmetry axis, which reflects a higher rigidity of the bilayer interior [26,34,35]. The rotational correlation time was calculated using the following equation:

(10) $$\tau =5.95\cdot △Bo\cdot \left(\sqrt{\frac{I_{0}}{I_{+1}}}+\sqrt{\frac{I_{0}}{I_{-1}}}-2\right)\cdot 10^{-10}[s]$$

where *I*_0_, *I*_−1_ and *I*_+1_ denote the amplitudes of the corresponding spectral lines, as illustrated in Figure 6a [26]. To highlight the relative changes caused by LPS incorporation, *τ* values were normalized by dividing by the initial value *τ*_0_, and expressed as *τ*/*τ*_0_.

**Figure 7:**
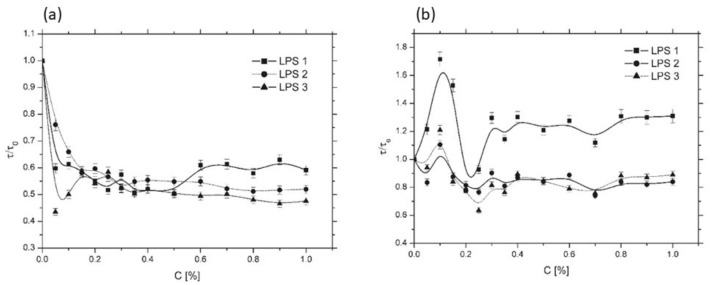
Influence of selected lipopolysaccharides (LPS) on the spectroscopic parameters of the 16-DOXYL stearic acid methyl ester spin probe incorporated into the lipid bilayer of liposomes composed of (**a**) egg yolk lecithin (EYL) and (**b**) dipalmitoylphosphatidylcholine (DPPC). The examined LPS samples were derived from *Hafnia alvei* PCM 1200 (LPS1), *Proteus penneri* 12 (LPS2), and *Proteus vulgaris* 9/57 (LPS3).

Figure 7a,b shows the influence of LPS admixtures on the *τ*/*τ*_0_ parameter for liposomes composed of egg yolk lecithin (EYL, Figure 7a) and dipalmitoylphosphatidylcholine (DPPC, Figure 7b). For EYL liposomes, whose membranes were in the liquid-crystalline phase, significant changes in *τ*/*τ*_0_ were observed for all LPS samples within the concentration range of 0–0.2%. Above 0.2%, *τ*/*τ*_0_ values stabilized at approximately 0.55. The strongest effect was found for LPS3. Admixtures of LPS1 and LPS2 produced nearly identical *τ*/*τ*_0_ profiles, suggesting that these molecules exerted a very similar influence on the fluidity of EYL liposomal membranes within the studied concentration range. In contrast, for DPPC liposomes, whose membranes were in the gel phase, the effect of LPS on *τ*/*τ*_0_ differed considerably. Although the most pronounced changes were again observed between 0 and 0.2%, their character varied. LPS2 and LPS3 exhibited a local maximum at 0.1% concentration with *τ*/*τ*_0_ ≈ 1.2 and 1.1, respectively, whereas LPS1 reached *τ*/*τ*_0_ ≈ 1.7, indicating a transient membrane rigidification at this concentration. Above concentrations 0.2%, LPS2 and LPS3 induced *τ*/*τ*_0_ values oscillating around 0.8, suggesting slight membrane fluidization, while LPS1 maintained *τ*/*τ*_0_ around 1.2, indicating persistent stiffening of the bilayer core. These observations confirm that both the structural state of the lipid matrix (liquid-crystalline vs. gel phase) and the specific type of LPS admixture significantly modulate the rotational dynamics of the spin probe, and consequently, the membrane fluidity. The results are consistent with previous EPR studies in which the 16-DOXYL spin probe served as a sensitive reporter of lipid bilayer dynamics and order in the hydrophobic region [34,35].

#### 3.1.2. Partition Coefficient Analysis

Figure 6b illustrates the dependence of the spectroscopic parameter, namely the partition coefficient (*F*), determined for the TEMPO spin probe localized within the hydrophobic region of the liposomal bilayer and in the surrounding aqueous phase. The molecular design of the probe positions its paramagnetic nitroxide moiety both in the water external to the liposome and within the membrane interior. An increase in the *F* coefficient indicates the probe’s preferential solubilization in the lipidic environment, reflecting enhanced fluidity within the bilayer core [26,34,35]. The partition coefficient was calculated according to the following equation:

(11) $$F=\frac{H}{\left(H+P\right)}\;$$

where *H* denotes the signal intensity of the probe in the lipid environment, and *P* represents the signal of the probe in the aqueous phase (Figure 6b). The TEMPO spin probe distributes between the lipid and aqueous phases in a ratio governed by the degree of membrane fluidity. An increase in membrane fluidity promotes the diffusion of the probe through the interfacial region into the lipid phase. Therefore, the TEMPO probe serves as an indirect reporter of the fluidity of the membrane surface region [36,37,38].

**Figure 8:**
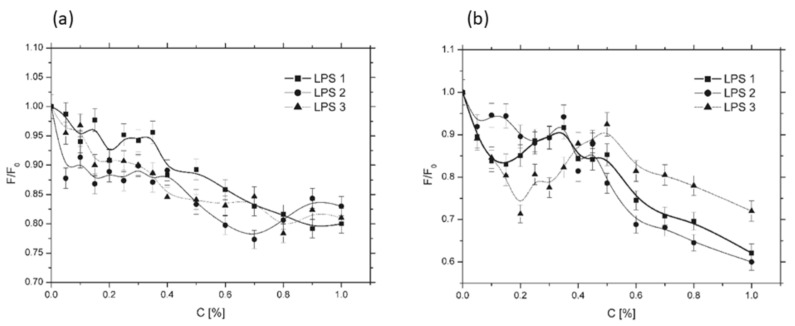
Influence of selected lipopolysaccharides (LPS) on the spectroscopic properties of the Tempo probe incorporated into the lipid bilayer of liposomes and the surrounding aqueous medium. Liposomes were composed of (**a**) egg yolk lecithin (EYL) and (**b**) dipalmitoylphosphatidylcholine (DPPC). The LPS samples examined originated from *Hafnia alvei* PCM 1200 (LPS1), *Proteus penneri* 12 (LPS2), and *Proteus vulgaris* 9/57 (LPS3).

Figure 8a,b illustrates the effect of LPS additives on the F/F_0_ ratio for liposomes composed of egg yolk lecithin (EYL, Figure 8a) and dipalmitoylphosphatidylcholine (DPPC, Figure 8b). For EYL liposomes, whose membranes were in the liquid-crystalline phase, a gradual decrease in the F/F_0_ ratio was observed for all LPS samples over the examined concentration range, indicating progressive membrane rigidification with increasing LPS content. At LPS concentrations above 0.8%, the changes tended to stabilize around an F/F_0_ value of approximately 0.82. In the case of DPPC liposomes, whose membranes were in the gel phase during the measurements, the effects of LPS additives were more variable, showing larger and more dynamic changes compared to EYL membranes. Within the LPS concentration range of 0–0.45%, dynamic fluctuations were observed. In the initial phase (0–0.15–0.2% LPS), LPS1 and LPS3 induced a pronounced decrease in F/F_0_, reaching local minima of approximately 0.85 and 0.75, respectively, indicating membrane stiffening. LPS2, however, did not exhibit this effect. At concentrations above 0.4%, all tested LPS additives caused a nearly monotonic decrease in F/F_0_, reflecting progressive membrane rigidification with increasing LPS content. The most pronounced changes in liposomal membrane fluidity were observed for LPS3.

### 3.2. Simulation Results

The main goal of the simulation was to investigate and compare with the EPR experiment the behavior of the membrane surface layer in the liquid crystal and gel phases at a temperature of 295 K under the influence of increasing concentrations of dopants representing the tested lipopolysaccharides (LPS). This was accomplished through bitmap analysis and calculation of the system’s binding energy. The dipoles were arranged in a rectangular 44 × 44 matrix. Based on the molecular structure of LPS, the hydrophilic moiety comprising lipid A and the inner core may introduce an electric charge into the membrane surface layer, which is associated with the phosphate groups present in the molecule. It is difficult to precisely determine the effective charge that can be introduced into the membrane; therefore, two charge values, *q* = 3 and *q* = 4, were assumed in the simulations, for which a complete set of concentration-dependent simulations was performed. The size of the molecule incorporated into the membrane was assumed to be 3 nm, based on previous studies [39,40]. The structure of the dipole array was modeled after the works of [25,41].

**Figure 9:**
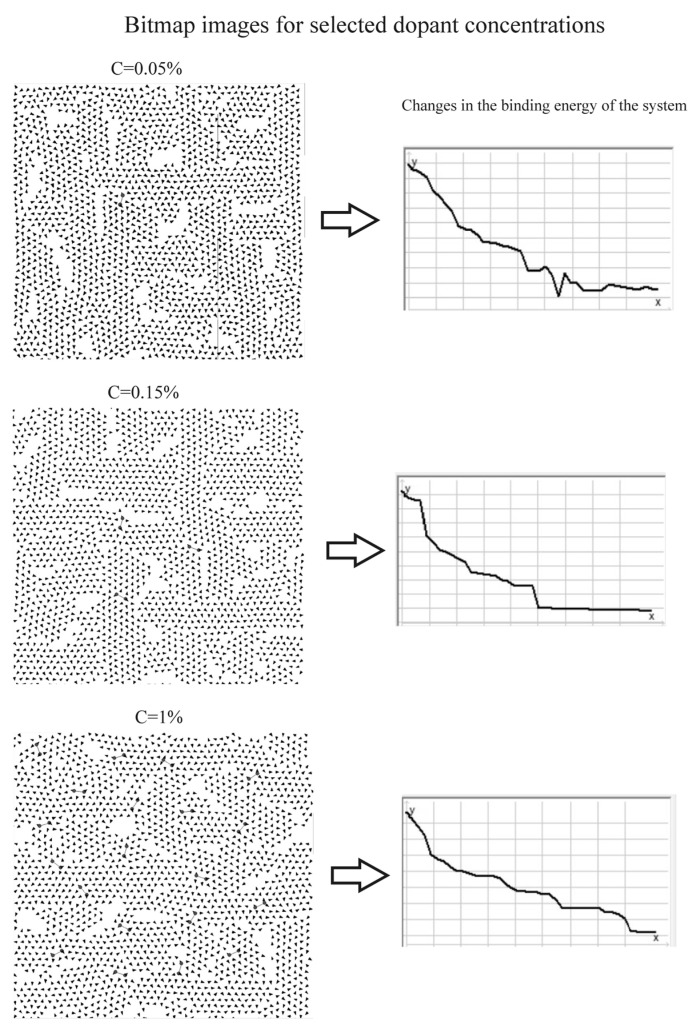
Representative bitmap images for rectangular arrays comprising 44 × 44 dipoles at selected dopant concentrations (C = 0.05%, C = 0.15%, and C = 1%) in the liquid crystal phase (*k* = 1). The corresponding graphs depict fluctuations in the binding energy as the system approaches equilibrium.

**Figure 10:**
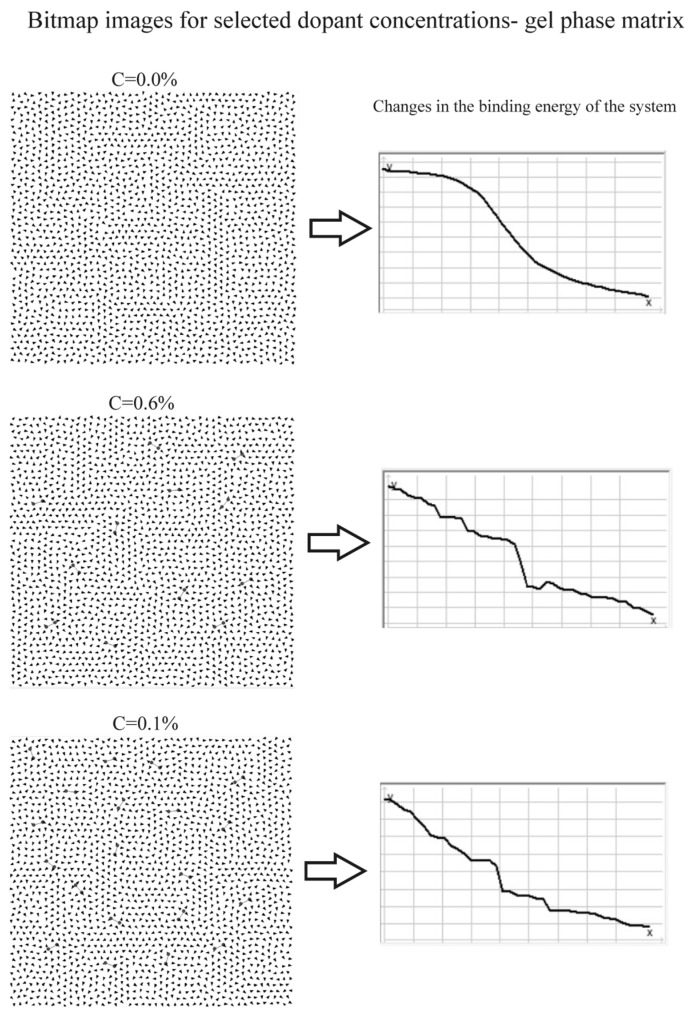
Representative bitmap images for rectangular arrays comprising 44 × 44 dipoles at selected dopant concentrations (C = 0.0%, C = 0.6%, and C = 1%) in the gel phase (*k* = 0.1). The corresponding graphs depict fluctuations in the binding energy as the system approaches equilibrium.

The initial dipole orientations were randomized using a pseudorandom number generator, after which dopants were introduced into the matrix in increasing concentrations: from 0 to 0.4% in steps of 0.05%, and from 0.4 to 1% in steps of 0.1%, relative to the total number of dipoles in the system. For each parameter set, five independent simulation runs were performed, and the results were averaged. The simulation software enabled real-time monitoring of dipole matrix rearrangements during subsequent simulation steps and tracked changes in the system’s binding energy, both numerically and graphically (Figure 9 and Figure 10).

#### 3.2.1. Bitmap Analysis

Bitmap analysis indicates that, in the liquid-crystalline phase, dipoles tend to organize into domains separated by defects, the number of which increases slightly with rising dopant concentration (Figure 9). The presence of these defects may facilitate enhanced penetration of the hydrophobic core by the aqueous environment. The system reaches equilibrium (minimum binding energy) in a jumping manner, suggesting the existence of local energy traps that can be destabilized by thermal fluctuations. In the gel phase, no significant defects in the dipole distribution within the matrices were observed (Figure 10). Although the formation of domain structures is evident, voids do not arise at their boundaries, in contrast to the liquid-crystalline phase. Increasing the dopant concentration did not induce defects in the dipole matrices. As in the liquid-crystalline phase, the system reached equilibrium in a jumping manner, through discrete changes in binding energy.

#### 3.2.2. Binding Energy Dependence on Dopant Concentration

**Figure 11:**
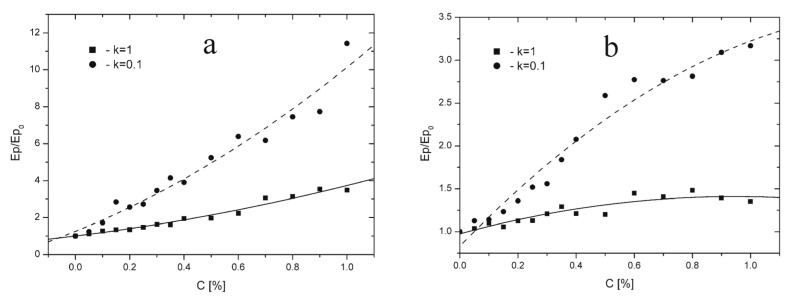
Influence of the concentration of selected dopants with length *L* = 3 nm, representing the tested LPSs, on the binding energy of a dipole system arranged in a 44 × 44 matrix for two matrix phases: the liquid-crystalline phase (*k* = 1) and the gel phase (*k* = 0.1). Results are shown for dopant charges: (**a**) *q* = 4*q* and (**b**) *q* = 3*q*.

To evaluate how different concentrations of dopants—characterized by physicochemical properties comparable to the LPS molecules tested experimentally—affect the dynamic properties of the membrane, a series of computer simulations was performed. The dopant concentration was varied from 0% to 1%, using a step size of 0.05% in the 0–0.4% range and 0.1% in the 0.4–1% range. Because LPS molecules may introduce different net charges into the membrane, charges of *q* = 4 and *q* = 3 were selected for the simulations. For clearer comparison of the outcomes, the simulation results were expressed as relative binding energies of systems at equilibrium by dividing the final energy at a given dopant concentration Ep/Ep_0_ by the final energy of the undoped system. The results are presented in Figure 11a,b.

The results of the computer simulations clearly indicate two fundamental parameters governing the influence of dopants on the binding energy of the system. The first is the phase state of the system. For *k* = 1, corresponding to high dipole mobility, the effect of dopants on the binding energy was substantially weaker than for *k* = 0.1, which reflects a pronounced stiffening of the system. This trend is evident in the slope of the curves with respect to the horizontal axis: in both Figure 11a,b, the curves corresponding to *k* = 0.1 are significantly steeper than those for *k* = 1. This result is somewhat unexpected and counterintuitive, as one might anticipate that the introduction of additional charge would more strongly disrupt the structure in systems with more mobile dipoles. This interpretation also appears consistent with the bitmap images shown in Figure 9 and Figure 10, where pronounced changes in dipole alignment are observed for *k* = 1, whereas for *k* = 0.1 such changes are considerably less apparent. This result can be explained by the fact that the gel-phase membrane models exhibit lower energetic and structural stability than those in the liquid crystalline phase. In the gel state, the arrangement of dipoles associated with the polar headgroups in the membrane surface layer is characterized by a lower absolute binding energy compared to an identical dipolar configuration in the liquid-crystalline state. Simulations performed for impurity-free dipolar lattices composed of 44 × 44 dipoles, representing the gel phase (*k* = 0.1) and the liquid-crystalline phase (*k* = 1), showed binding energies of −14,531 a.u. for the gel phase and −14,201.5 a.u. for the liquid-crystalline phase. These results indicate stronger dipole–dipole interactions and enhanced energetic stability of the liquid-crystalline membrane.

**Figure 12:**
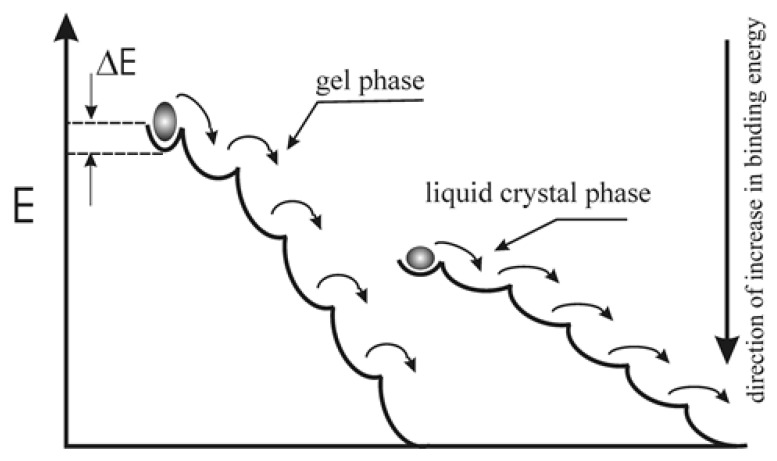
Schematic representation of the system evolution from the initial to the final state, illustrating two scenarios corresponding to the membrane in the gel phase and in the liquid-crystalline phase, respectively.

The reduced binding energy observed in the gel phase suggests an increased susceptibility of this state to impurity incorporation. Such perturbations may induce pronounced energetic rearrangements, facilitating transitions toward more stable configurations characterized by higher binding energies. This concept can be illustrated schematically in Figure 12. The curves represent the system’s evolution toward a maximum binding energy; the steeper curve corresponds to the dipolar system in the gel phase, whereas the less steep curve represents the liquid-crystalline phase. The value ΔE denotes the energy trap in which the system resides at a given simulation stage.

The transition of the system from the initial to the final state proceeds through successive energy traps, illustrated by a ball rolling downhill. Each trap corresponds to a metastable state of the system. The temperature at which the system operates acts as a perturbing factor, enabling the system to overcome successive energy barriers and ultimately reach a state of minimum binding energy, which corresponds to the final state of the simulation for a given set of input parameters. In real biological membranes, a similar scenario is likely to occur within the surface layer, where polar headgroups may behave analogously to the electric dipole lattices investigated in this study.

The second key parameter is the magnitude of the charge introduced into the matrix. Reducing the dopant charge from *q* = 4 to *q* = 3 markedly diminished its effect on the binding energy at comparable concentrations. For instance, at C = 0.8%, the Ep/Ep_0_ values for a matrix doped with *q* = 4 were approximately 3 for *k* = 1 and around 8 for *k* = 0.1. When the matrix was doped with *q* = 3, the corresponding values were roughly 1.4 for *k* = 1 and 2.75 for *k* = 0.1. These findings may have practical implications, suggesting the possibility of designing controlled modifications of membrane fluidity through the selection of macromolecules that contribute charge to the surface layer.

Further conclusions can be drawn from the analysis of charge effects. The experimental data suggest that the effective charge modeling the surface layer of liposomes is closer to *q* = 3 than to *q* = 4. The dynamics of changes in the F/F_0_ parameter are consistent with the dynamics of the binding energy obtained for a dopant with *q* = 3, as evidenced by the shape of the curves. In Figure 8a, we observe only a slight change in F/F_0_ as a function of concentration; after an initial doping phase characterized by somewhat higher dynamics, the curve gradually reaches a plateau with increasing dopant concentration.

A similar behavior can be observed for the curve shown in Figure 11b for *k* = 1, representing the model of the membrane surface layer in the liquid-crystalline phase. Here, we also observe only small changes in the binding energy Ep/Ep_0_ as a function of the concentration of a dopant with charge *q* = 3. Changes in this energy correspond to changes in membrane fluidity in real membranes, since an increase in Ep/Ep_0_ indicates an increase in binding strength between system components and, consequently, an increase in membrane rigidity. In the computational model, Ep = 0 corresponds to a situation in which the membrane is dissolved in the surrounding medium (i.e., complete destruction of the structure). An increase in the absolute value of this energy reflects stronger interactions between system components and, therefore, membrane stiffening.

An analogous situation is observed when comparing the dynamics of the curves shown in Figure 8b and Figure 11b for *k* = 0.1. We observe a systematic change in the Ep/Ep_0_ parameter (Figure 8b) as a function of concentration, particularly pronounced above 0.5%, which corresponds well with the changes observed in Figure 11b for *k* = 0.1.

In contrast, the analysis of the plots shown in Figure 11a, representing a system doped with a species of charge *q* = 4, reveals a monotonic increase in Ep/Ep_0_ for both *k* = 1 and *k* = 0.1. In this case, the curve for *k* = 0.1 increases very rapidly with increasing dopant concentration, which is inconsistent with the experimental measurements. Such pronounced changes in the F/F_0_ parameter were not observed for membranes in the gel phase. Moreover, the Ep/Ep_0_ dependence for membranes in the liquid-crystalline phase is also inconsistent with experimental data. The curve for *k* = 1 likewise shows a continuous increase in Ep/Ep_0_ with increasing concentration, which does not agree with the experimental observations. When analyzing these plots, it should be noted that a decrease in the F/F_0_ partition parameter is associated with an increase in membrane rigidity. Therefore, the curves in Figure 8, approaching the C [%] axis, represent increasing rigidity, whereas in the case of binding energy Ep/Ep_0_ versus dopant concentration (Figure 11), the situation is reversed. As the binding energy increases, the rigidity of the system increases and membrane fluidity decreases; consequently, the curves move away from the C [%] axis. In contrast, LPS admixtures influenced the interior of the lipid bilayer (the hydrophobic region) only at low concentrations—0.1% for EYL membranes (Figure 7) and 0.2% for DPPC membranes (Figure 7b). Above these concentrations, the observed changes were minimal. Notably, in this case, the LPS admixtures affected EYL membranes more strongly, i.e., membranes in the liquid-crystalline state. This effect may be associated with the formation of a greater number of defects in the surface layer of these membranes, as suggested by Figure 9 (bitmap maps from the simulations).

## 4. Conclusions

The aim of this study was to assess whether macromolecules of selected LPSs, to varying degrees, affect the dynamic properties of liposomal membranes in the liquid-crystalline and gel phases, and whether this influence can be predicted through computer simulations of their surface layers. The results demonstrated a strong correlation between the computational model and the spin-probe experiments. Both approaches consistently indicated that LPS admixtures exert a more pronounced effect on membrane fluidity in the gel phase. The TEMPO probe, which accurately reflects alterations within the membrane surface layer [2,42,43], revealed a substantially stronger impact on DPPC liposomal membranes (gel phase) compared with EYL membranes (liquid-crystalline phase). Correspondingly, the partition coefficient F/F_0_ reached lower values in DPPC membranes than in EYL membranes, in agreement with the simulation outcomes.

Although the overall structures of the tested LPS molecules were similar, their effects on the dynamic properties of liposomal membranes were not identical. This observation indicates that the hydrophilic portion of the LPS molecules may also contribute to modulating membrane rigidity. The specific influence of the hydrophilic moiety of LPS on the fluidity of liposomal lipid bilayers requires further investigation using complementary analytical techniques to enable definitive conclusions.

## Data Availability

The original contributions presented in the study are included in the article. Further inquiries can be directed to the corresponding author.
